# LIN28 Expression in Rat Spinal Cord After Injury

**DOI:** 10.1007/s11064-014-1278-2

**Published:** 2014-04-04

**Authors:** Ying Yue, Dongmei Zhang, Shengyang Jiang, Aihong Li, Aisong Guo, Xinming Wu, Xiaopeng Xia, Hongbing Cheng, Tao Tao, Xingxing Gu

**Affiliations:** 1 The Jiangsu Key Laboratory of Neuroregeneration, Nantong University, Nantong, 226001 People’s Republic of China; 2 Department of Pathogen Biology, Medical School, Nantong University, Nantong, 226001 People’s Republic of China; 3 Basic Medial Research Center, Medical School, Nantong University, Nantong, 226001 People’s Republic of China; 4 Department of Neurology, Affiliated Hospital of Nantong University, Nantong, 226001 People’s Republic of China; 5 Department of Orthopaedics, Traditional Chinese Medical Hospital of Nantong City, Nantong, 226001 People’s Republic of China; 6 Department of Chemistry and Institutes of Biomedical Sciences, Fudan University, Shanghai, 200433 People’s Republic of China

**Keywords:** LIN28, Spinal cord injury, Inflammatory, Astrocytes

## Abstract

LIN28, an RNA-binding protein, is known to be involved in the regulation of many cellular processes, such as embryonic stem cell proliferation, cell fate succession, developmental timing, and oncogenesis. However, its expression and function in central nervous system still unclear. In this study, we performed an acute spinal cord contusion injury (SCI) model in adult rats and investigated the dynamic changes of LIN28 expression in spinal cord. Western blot and immunohistochemistry analysis revealed that LIN28 was present in normal spinal cord. It gradually increased, reached a peak at 3 day, and then nearly declined to the basal level at 14 days after SCI. Double immunofluorescence staining showed that LIN28 immunoreactivity was found in neurons, astrocytes and a handful of microglia. Interestingly, LIN28 expression was increased predominantly in astrocytes but not in neurons. Moreover, the colocalization of LIN28 and proliferating cell nuclear antigen was detected after injury. Western blot showed that LIN28 participated in lipopolysaccharide (LPS) induced astrocytes inflammatory responses by NF-κB signaling pathway. These results suggested that LIN28 may be involved in the pathologic process of SCI, and further research is needed to have a good understanding of its function and mechanism.

## Introduction

Spinal cord injury (SCI) is a devastating and common neurologic disorder that has profound influences on modern society from physical, psychosocial, and socioeconomic perspectives [1–3]. It is generally accepted that acute spinal cord injury (SCI) is a two step process involving primary and secondary injury mechanisms [4, 5]. Secondary injury of SCI may result from spinal cord edema, ischemia, free radical damage, electrolyte imbalance, excitotoxicity, inflammatory injury, and apoptosis [6–9]. These factors cause astrocytes proliferation and reactive gliosis, resulting in the formation of a dense astrocytic scar [10, 11]. This glial scar provides a physical and biochemical barrier to regeneration and plasticity, and acts as a source of multiple inhibitory factors that affect functional recovery from SCI [12, 13]. All these alterations contribute to the long term neurological damage which results in neuronal loss and causes a variety of neurological, behavioral, emotional, and cognitive deficits [9]. An increasing number of researches have implicated neurological damage and dysfunction after SCI [5, 14]. However, it is remain to be further elucidated about the molecular mechanisms of post traumatic pathology of spinal cord.

The heterochronic gene, LIN28, is an evolutionarily conserved RNA-binding protein that was first identified in the nematode *Caenorhabditis elegans*, in which it displays tissue- and stage-specific patterns of expression. LIN28 mutations results in either the advanced or delayed maturational events [15, 16]. The mammalian genome encodes two homologs of the *C. elegans* LIN28 genes, LIN28A and LIN28B [17, 18]. In mammals, LIN28, also termed LIN28A, is ubiquitously expressed in early embryonic stages. As development proceeds, its Multi-expressed in several tissues such as cardiac and skeletal muscles [19, 20]. With research on Lin28 growing number of studies have found it also expressed in the central nervous system [21]. This is consistent with the results we saw in the expression of Lin28 in GeneCards. LIN28 is known to be involved in many important processes such as germ cell development [22], embryogenesis [23], skeletal myogenesis [24],glucose metabolism [25], cell fate succession [26, 27], and cellular differentiation [28, 29].

Extensively expressed in embryonic stem (ES) and embryonic carcinoma (EC) cells, LIN28 promotes pluripotency and proliferation in these cells by regulating let-7 family microRNAs (miRNAs) or acting independently of miRNAs [30–33]. Besides its role on pluripotency, LIN28 is known to regulate the translation of a set of genes crucial for the growth and survival of human ES cells [33]. In agreement with this, [9] reported the pro-growth function of LIN28 in mouse primary hippocampal neurons. Moreover, it has been reported that LIN28 may be associated with the enhanced viability of cancer and ES cells [34, 35]. Based on these above results, it seems that LIN28 may play a formerly unknown role in the regulation of cellular processes. However, the physiological role of LIN28 in central nervous system (CNS) remains to be investigated.

In this study, we examined the temporospatial expression of LIN28 protein and its colocalization with proliferating cell nuclear antigen (PCNA) in an acute SCI model of adult rats. We also found LIN28 participated in astrocytes inflammatory responses through NF-κB signaling pathway. These data were conducted to gain greater insight into the functions of LIN28 and its roles in the cellular and molecular mechanisms underlying central nerve injury and repair.

## Materials and Methods

### Animals

Adult male Sprague–Dawley rats weighing 200–250 g (Experimental Animal Center, Nantong University, China) were used in this study. All protocols with animals were approved by the Institutional Animal Care and Use Committee in accordance with the National Institutes of Health Guide for the Care and Use of Laboratory Animals (1996) and guidelines for the International Association for the Study of Pain [36] and were conducted according to the Animals Care and Use Committee of Nantong University and approved by the Jiangsu Province Animal Care Ethics Committee.

### Behavior Analysis

The Basso, Beattie, and Bresnahan (BBB) open field score was used to assess locomotion in terms of hind limb functional improvement of rats with spinal cord contusion. The BBB test was scored from 0 (no observable hind limb movement) to 21 points (normal coordinate gait), using paw placement, joint movement, and truncal stability as important factors in determining the level of functional recovery. Scores in the 0–7 range focus primarily on hip, knee, and ankle joint movement, the 8–13 range keys in on paw placement and coordination, and scores of 14–21 rely heavily on trunk stability, tail position, and paw placement. In this study, the behavior analysis was performed at 0, 6, 12 h, 1, 3, 5, 7 and 14 days after injury. The results are presented as mean ± S.E. The two tailed Mann–Whitney test and repeated measures analysis of variance were performed to evaluate the statistical significance of the results using SPSS 11.0 software. The *p* value obtained from these method was <0.05.

### Surgical Procedures

Dorsal laminectomies at the level of the ninth thoracic vertebra (T9) were carried out under anesthesia with ketamine (90 mg/kg)/xyla-zine (10 mg/kg), and surgery was performed in aseptic conditions. Ketoprofen (5 mg/kg) was administered to minimize postsurgical pain and discomfort. Contusion injury groups (n = 51) were performed using the NYU impactor [37]; the exposed spinal cord was contused by dropping a rod 2.0 mm in diameter and 10 g in weight from a height of 75 mm for 33 rats [38–40]. The rest of the contusion injuries (n = 21) were divided randomly into seven groups of three animals. The seven groups were contused from different heights (6.25, 1 2.5, 25, 50, 75, 100, and 125 mm, respectively). Sham operated animals (n = 12) were anesthetized and surgically prepared but did not receive spinal injury. After SCI, the overlying muscles and skin were closed in layers with 4–0 silk sutures and staples. The animals were allowed to recover on a 30 °C heating pad. Postoperative treatments included saline (2.0 ml, s.c.) for rehydration and Baytril (0.3 ml, 22.7 mg/ml, s.c. once daily) to prevent urinary tract infection. Bladders were manually expressed twice daily until reflex bladder emptying returned. Animals were housed under a 12 h light/dark cycle in a pathogen-free area with free access to water and food. The spontaneous recovery of locomotor function after SCI was examined using the Basso, Beattie and B resnahan (BBB) rating scale [41]. Animals were killed at 6, 12 h, 1, 3, 5, 7, and 14 days after injury. Fifteen sham animals were used as sham controls. All efforts were made to minimize the number of animals used and their suffering.

### Primary Astrocyte Cultures and Cell Treatment

Astrocyte cultures were prepared from spinal cords of adult male Sprague–Dawley rats using a previously described method [42, 43] with a few modifications. The spinal cords were ejected from the vertebral column using a saline-filled syringe. The tissue was chemically dissociated with 0.25 % trypsin–EDTA for 10 min followed by mechanical trituration in modified essential medium (DMEM). After centrifugation at 1,500 rpm for 5 min, the cells were suspended with DMEM/F12 culture medium, containing 10 % heat-inactivated FBS and 1 % glutamine, and plated in a flask. The cultures were maintained in a humidified atmosphere of 95 % O_2_/5 % CO_2_ at 37 °C for 48–72 h to allow the cells sufficient time to adhere and begin multiplying. The medium was changed at every 24 h. Approximately on days 10 and 11, flasks were wrapped in plastic, placed on a shaker platform in a horizontal position with the medium covering the cells, and were shaken at 350 rpm for 6 h at 37 °C to separate the microglia from the astrocytes. The next day, the cells were trypsinized and replanted in six-well plates (40,000 cells per well). Prior to experimental treatments, cultures of astrocytes were passaged twice. Cell culture medium was switched to serum-free DMEM/F12 culture medium.

The astrocytes were synchronized for 24 h in the absence of serum and then incubated in the absence of serum and in the presence or absence of 1 μg/ml lipopolysaccharide (LPS). Three different treatment regimens were assigned: (1) untreated controls, (2) cells treated with LPS (1 μg/ml), (3) cells pretreated with PDTC (100 μM) for 2 h and treated with LPS (1 μg/ml). Then these cells were harvested for western blot analysis.

### Western Blot

In order to obtain samples for western blot analysis, the sham or injured spinal cords were excised. The portion of the spinal cord extending 5 mm rostral and 5 mm caudal to the injury epicenter was dissected out and immediately frozen at −80 °C until use. To prepare lysates, frozen spinal cord samples were minced with eye scissors on ice. The samples were then homogenized in lysis buffer (1 % NP-40, 50 mmol/L Tris, pH 7.5, 5 mmol/L EDTA, 1 % SDS, 1 % sodium deoxycholate, 1 % Triton X-100, 1 mmol/L PMSF, 10 mg/ml aprotinin, and 1 mg/ml leupeptin) and clarified by centrifuging for 20 min in a microcentrifuge at 4 °C. After determination of its protein concentration with the Bradford assay (Bio-Rad), the resulting supernatant (50 μg of protein) was subjected to SDS–polyacrylamide gel electrophoresis (10 % of acrylamide). The separated proteins were transferred to a polyvinylidene difluoride membrane (Millipore) by a transfer apparatus at 300 mA for 2 h. The membrane was then blocked with 5 % nonfat milk and incubated with primary antibodies against LIN28 (anti-rabbit, 1:600; Abcam), GAPDH (anti-rabbit, 1:1000; Sigma), NF-κB p50 (anti-rabbit, 1:500; Sigma), NF-κB p65 (anti-rabbit, 1:1000; Sigma), p-p65 (anti-rabbit, 1:800; Abcam), or p-IκBα(anti-rabbit, 1:500; Abcam) at 4 °C overnight. After incubating with horseradish peroxidase-conjugated secondary antibody, protein was visualized using an enhanced chemiluminescence system (Pierce Company, USA).

### Immunohistochemistry, Immunofluorescent, and H&E Staining

The sham and injured rats were terminally anesthetized and perfused through the ascending aorta with saline, followed by 4 % paraformaldehyde at different survival time points. After the perfusion, the spinal cords of the sham and injured rats were removed and post-fixed in 4 % paraformaldehyde for 3 h. The spinal cords were then subsequently placed in 20 % sucrose for 2–3 days and followed by 30 % sucrose for an additional 2–3 days. Next, the spinal cords were embedded in O.T.C. compound for cryosectioning. The tissue was transversely cut into 9-μm-thick sections at two spinal cord levels (3 mm rostral and caudal to the epicenter of the injury) and examined. All of the sections were blocked with 10 % donkey serum with 0.3 % Triton X-100 and 1 % (w/v) bovine serum albumin (BSA) for 2 h at room temperature (RT) and was incubated overnight at 4 °C with anti-LIN28 (anti-rabbit, 1:100; Abcam), followed by incubation in a biotinylated secondary antibody (Vector Laboratories, Burlin-game, CA). The immunostaining was visualized with DAB (Vector Laboratories). Cells with strong or moderate brown staining were counted as positive, cells with no staining were counted as negative, and the cells with weak staining were scored separately.

For hematoxylin and eosin (H&E) staining, spinal cord samples were fixed in 10 % formalin solution, embedded in paraffin,and sliced into cross-sections of 7-μm thickness at two spinal cord levels (2 mm rostral and caudal to the epicenter of injury). Then, these sections were stained with hematoxylin and eosin.

For double immunofluorescent staining, slide-mounted sections were removed from the freezer and incubated in an oven at 37 °C for 40 min. Next, the sections were incubated in a blocking solution, containing 10 % normal serum from the same species as the secondary antibody, 3 % BSA, 0.1 % Triton X-100 and 0.05 % Tween-20 for 2 h at RT to prevent nonspecific staining. Next, the sections were incubated with anti-LIN28 (anti-rabbit, 1:100; Abcam). The cell-specific markers NeuN (neuronal marker, 1:100; Millipore, MAB377), GFAP (astrocytic marker, 1:100; Sigma), S100B(astrocytes marker, 1:100; Sigma), Iba1(microglia marker, 1:50; Serotec) or proliferating cell nuclear antigen (PCNA, anti-mouse, 1:50; Santa Cruz) were applied simultaneously. The sections were incubated with the primary antibodies overnight at 4 °C, followed by a mixture of CY2- and CY3-conjugated secondary antibodies for 2 h at 4 °C. The stained sections were then examined using a Leica fluorescence microscope (Germany).

### Quantitative Analysis

The number of LIN28-positive cells in the spinal cord 2 mm rostral to the epicenter was counted in a 500 × 500 μm measuring frame. For each animal, a measure was taken in a section through the dorsal horn, the lateral funiculus, and the ventral horn. We counted every fifth section (50 μm apart) to avoid counting the same cell in more than one section. The cell counts were then used to determine the total number of LIN28-positive cells per square millimeter. We simultaneously quantified the percentage of cells with cytoplasmic LIN28 staining. Cells double-labeled for LIN28 and the cell-specific makers NeuN, GFAP, S100B, Iba1 and PCNA were quantified, too. In order to identify the proportion of each phenotype-specific marker-positive cells expressing LIN28, a minimum of 200 phenotype specific marker-positive cells were counted in both the white matter and gray matter in each section. We then recorded the number of cells double-labeled with LIN28 and a cell-specific marker. Two or three adjacent sections per animal were sampled at 2 mm from the epicenter.

### Statistical Analysis

All of the data were analyzed with the Stata 7.0 statistical software. All of the values were expressed as the mean ± SEM. One-way ANOVA followed by Tukey’s post-hocmultiple comparison tests was used for the statistical analysis. *p* values less than 0.05 were considered statistically significant. Each experiment consisted of at least three replicates per condition.

## Results

### Behavioral Changes, Histopathological and Morphological Examination Following Traumatic Spinal Cord Injury

**Fig. 1:**
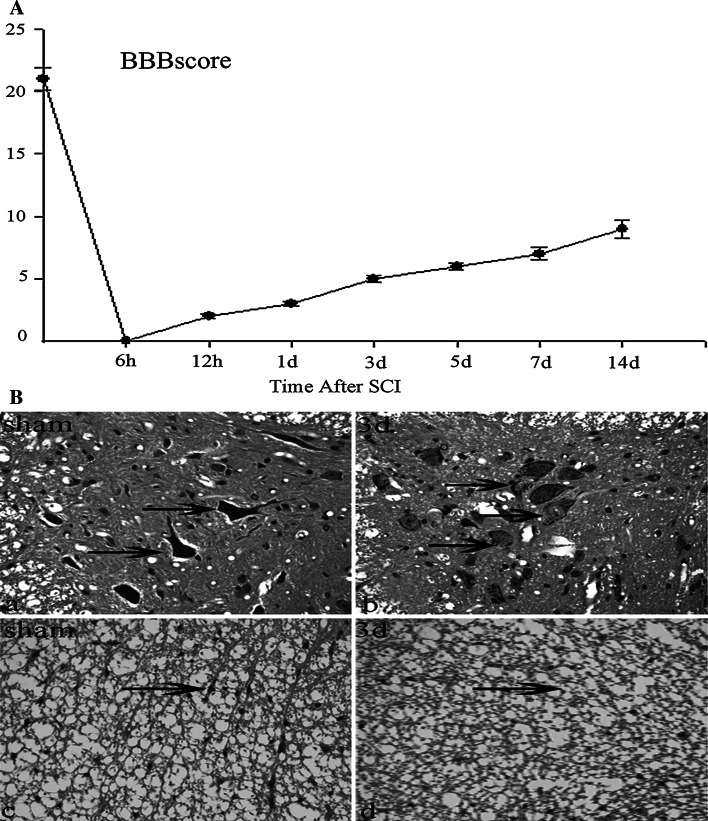
Behavior analysis and histology of the spinal cord of experimental animals. **a** Time course and degree of functional recovery in rats after SCI. Rats (n = 3) were killed at each time point (at 6 h and 12 h and at 1, 3, 5, 7, and 14 days post-injury). Data are reported as mean ± values of open-field locomotion BBB scores. **b** Histopathologic analysis of spinal cord from sham and injured rats at day 3 was used by H&E staining with paraffin histopathology sections. No lesion was seen in the sham group (*a*, *c*). Edema, vacuoles, irregularly shaped spaces, axon degradation, and disorders of the organization were evident in white matter at day 3 after injury (*arrows* in *d*). The majority of typical characteristics of neuronal apoptosis including nuclear fragmentation, nuclear disappearance, and nuclear pyknosis were found in gray matter (*arrows* in *b*). *Scale bars*, 20 μm (*a*–*d*)

The spontaneous recovery of locomotor function after spinal cord injury was tested by using the BBB rating scale [41]. Scores were recorded and the averages are shown in (Fig. 1). All animals slacked hindlimb locomotion after contusion to their spinal cords. Some spontaneous improvement in function occurred with time after SCI.

Then to analyze histology and morphology after spinal cord injury, we performed H&E staining for spinal cord with paraffin histopathology sections. Vacuoles, Edema, irregularly shaped spaces, axon degradation, and disorders of the organization in the white matter were noted in the areas extending 2 mm from the center of the SCI lesions (Fig. 1d), while no lesions were observed in the sham controls (Fig. 1a, c). The majority of typical characteristics of neuronal apoptosis including nuclear fragmentation, nuclear disappear,and nuclear pyknosis were seen in the gray matter (Fig. 1b).

### The Temporal Expression of LIN28 Following Spinal Cord Injury

**Fig. 2:**
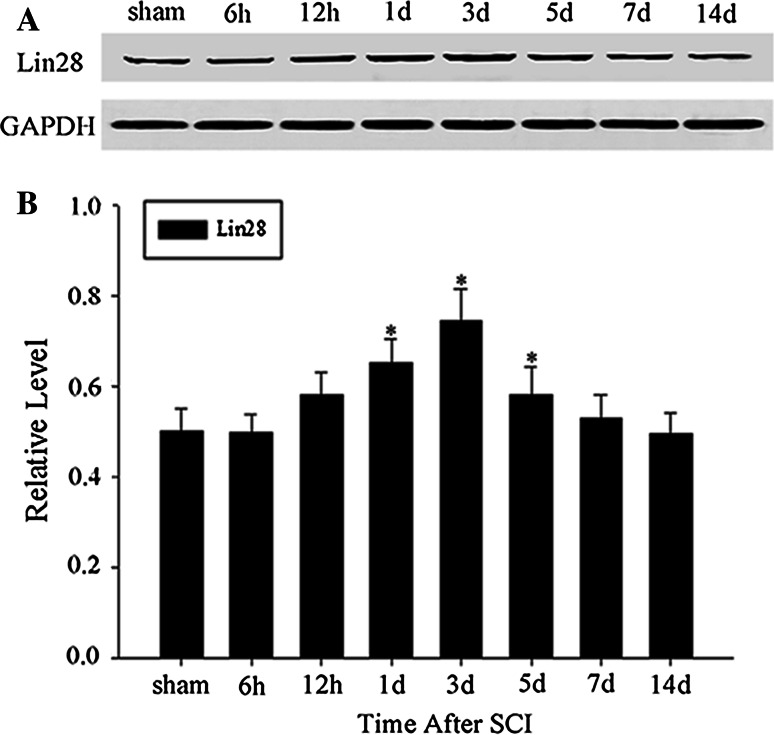
Time-dependent expression of LIN28 protein in rat spinal cord after SCI. Spinal cord tissues from rats at various survival times after SCI were homogenized and subjected to immunoblot analysis. Sample immunoblots probed with LIN28 and GAPDH are shown above (**a**). The *bar chart* below demonstrates the ratio of LIN28 to GAPDH for each time point (**b**). Data are mean ± SEM (n = 3; *p < 0.05, significantly different from the sham groups)

We examined the temporal expression patterns of LIN28 following spinal cord contusion injury by western blot. It was proved that LIN28 expression was low in sham operated spinal cords but increased at 12 h after SCI and reached a peak at day 3 (*p < 0.05); then, it gradually decreased to control levels (Fig. 2a, b). This result demonstrated that LIN28 might be involved in the process of SCI in a time dependent manner.

### Dose–Response Effects’ Between the SCI and LIN28 Expression

**Fig. 3:**
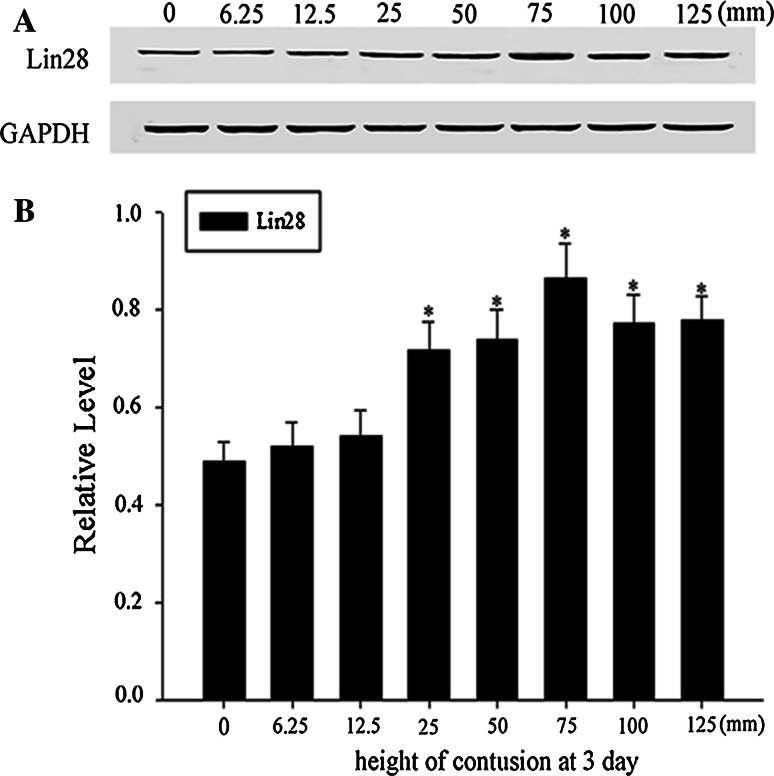
Dose-response effects’ between the SCI and LIN28 expression. Spinal cord tissues from rats at various degrees of contusion induced by different heights (0, 6.25, 12.5, 25, 50, 75, 100, and 125 mm) at day 1 after SCI were homogenized and subjected to immunoblot analysis. Sample immunoblots probed with LIN28 and GAPDH are shown above (**a**). The *bar chart* below demonstrates the ratio of LIN28 to GAPDH for each height point (**b**). Data are mean ± SEM (n = 3; *p < 0.05, significantly different from the sham group)

To further testify that LIN28 expression is related to the SCI, we investigated the effects of contusion induced by dropping the rod from different heights (0, 6.25, 12.5, 25,50, 75, 100, and 125 mm) at day 3 after SCI and observed the expression patterns of LIN28 by western blot. Following the contusion height being increased, LIN28 expression enhanced gradually, got a peak at 75 mm height, and then declined weakly in the case of 100 and 125 mm injury. However, contusion induced by 6.25 and 12.5 mm height could not raise the up-regulation of LIN28 expression, compared with its expression in the sham groups (Fig. 3a, b). These data demonstrated that the up-regulation of LIN28 is indeed related to spinal cord injury.

### Changes in Expression and Distribution of LIN28 in the Spinal Cord

**Fig. 4:**
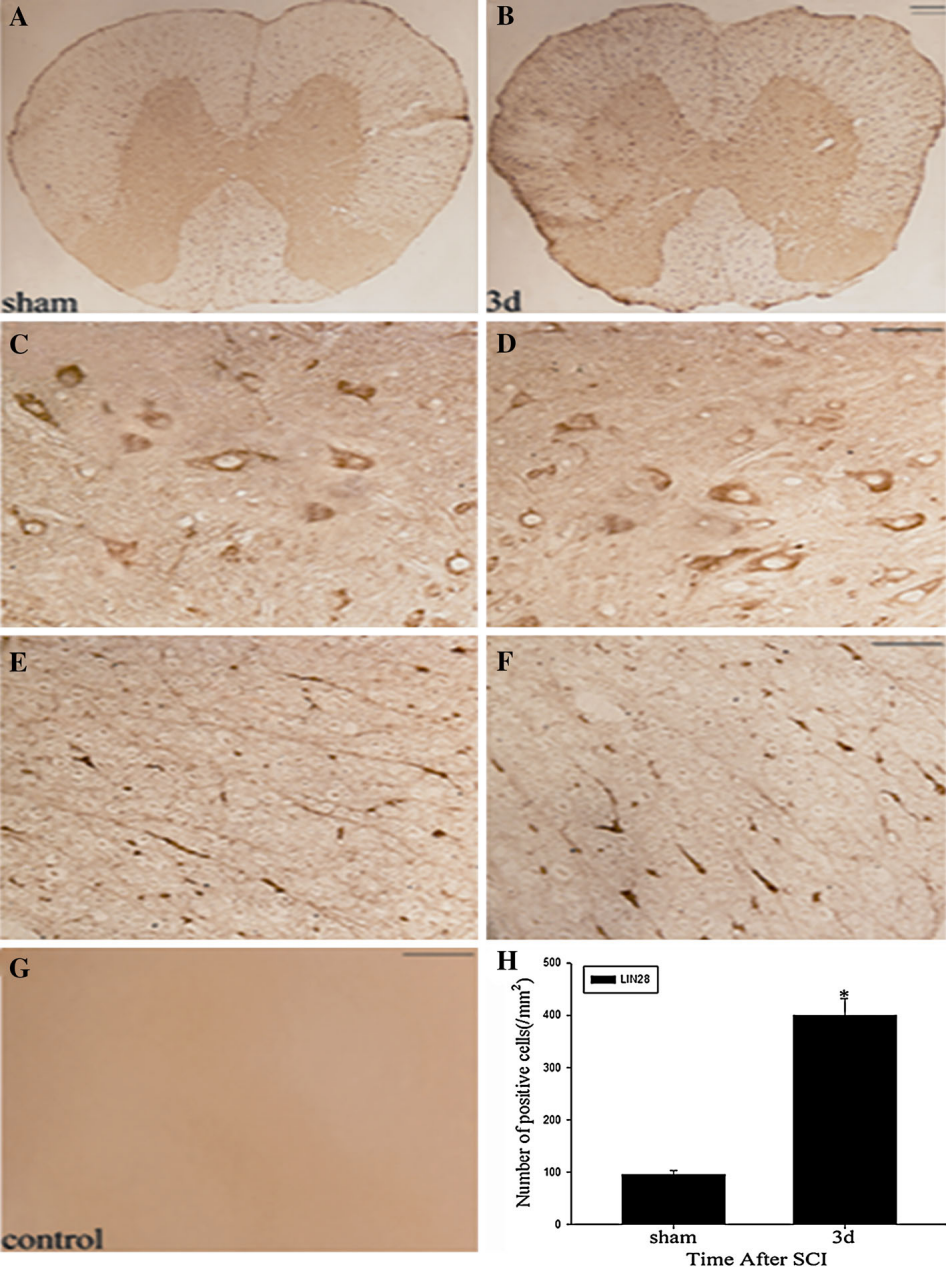
Immunohistochemical expression of LIN28 in adult rat spinal cord. Low-power views of cross-sections immunostained with antibody specific for LIN28 in sham spinal cord (**a**) and 3 day after injury (**b**). Higher-power views in the ventral horn (**c**, **d**) and white matter (**e**, **f**) of the spinal cord. Quantitative analysis of LIN28-positive cells/mm^2^ between sham and at day 3 after SCI (**g**). *p < 0.05 compared with sham spinal cord. *Error bars* represent SEM. *Scale bars*, 200 μm (**a**, **b**) and 20 μm (**c**–**g**)

To identify the cellular localization and the temporal changes of LIN28 immunoreactivity in spinal cord, we carried out immunohistochemisry experiments with anti-LIN28 rabbit polyclonal antibody on transverse cryosections of the spinal cord, 2 mm rostral to the epicenter. As shown above, LIN28 protein has the maximal protein expression at day 3. Thus, we chose 3 days after SCI as the time point for our microscopic work. According to the positive cell profiles, LIN28 was widely expressed in both the ventral horn and white matter (Fig. 4a, b) including neurons and glial cells, regardless of whether sham or injury. Interesting, at high magnification, injury increased LIN28 significantly in white matter (Fig. 4e, f), but the positively stained intensity in gray matter did not change obviously (Fig. 4c, d). No staining was observed in the negative control sections (Fig. 4g). At the same time, we found their quantitative changes to be parallel with the western blot results (Fig. 4h).

### The Colocalization of LIN28 with Different Phenotype Specific Markers in the Adult Rat Spinal Cord After SCI

**Fig. 5:**
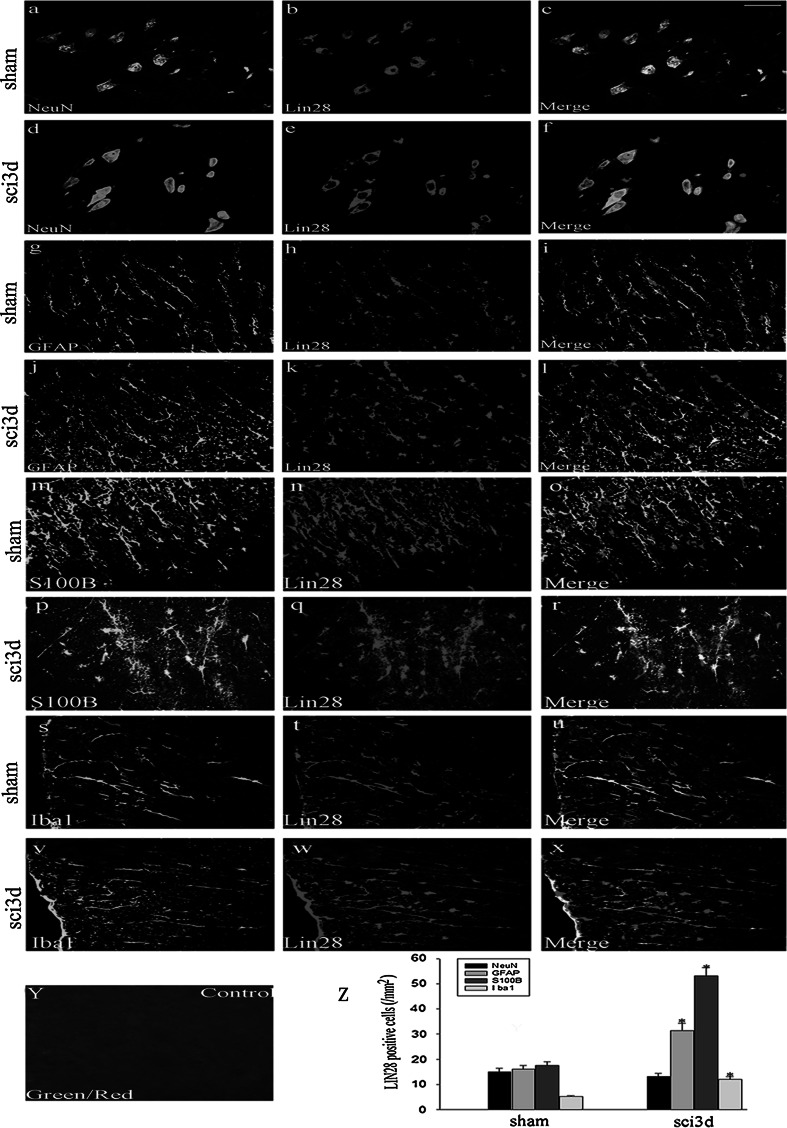
Double immunofluorescence staining for LIN28 and different phenotype-specific markers in the spinal cord. In the adult rat spinal cord 2 mm to epicenter at day 3 after SCI, horizontal sections were labeled with LIN28 (*red*) and different phenotype-specific markers (*green*), such as NeuN, GFAP, S100B and Iba1. The colocalization of LIN28 with different phenotype specific markers are shown in **c**, **f**, **i**, **l**, **o**, **r**, **u**, **x** and negative control in **y**. Quantitative analysis of different phenotype-specific markers positive cells expressing LIN28 (/mm^2^) in sham spinal cord and at 3 day after SCI (*p < 0.05, the changes of LIN28 expression after SCI were clear in astrocytes, but not in neurons) compared with the sham group (**z**). *Error bars* represent SEM. *Scale bars*, 20 μm (**a**–**m**) (Color figure online)

To further address the expression of LIN28 in the spinal cord, we performed double immunofluorescent microscopy studies in transverse cryosections of spinal cord tissues within 2 mm distance from the lesion site by colabeling with NeuN, GFAP, S100B or Iba1. We found LIN28 expression in neurons, astrocytes and few microglia (Fig. 5). Significantly, such expression was increased more significantly in astrocytes at 3 day after SCI compared with the sham spinal cord (Fig. 5g–l). To further confirm this result, we have chosen S100B which a functional astroglial marker, also found that the above phenomena (Fig. 5m–r). LIN28 is expressed in microglia also change but the expression is far less than astrocytes, so we focused on studies of it relationship with the astrocytes (Fig. 5s–x). No staining was observed in the negative control sections (Fig. 5y). As shown in (Fig. 5z), the numbers of astrocytes and LIN28-positive astrocytes increased prominently after injury compared with sham-operated spinal cord (*p < 0.05), while such change was not detected in neurons.

### Colocolization of LIN28 and the Cellular Proliferation Marker in the Adult Rat Spinal Cord after SCI

**Fig. 6:**
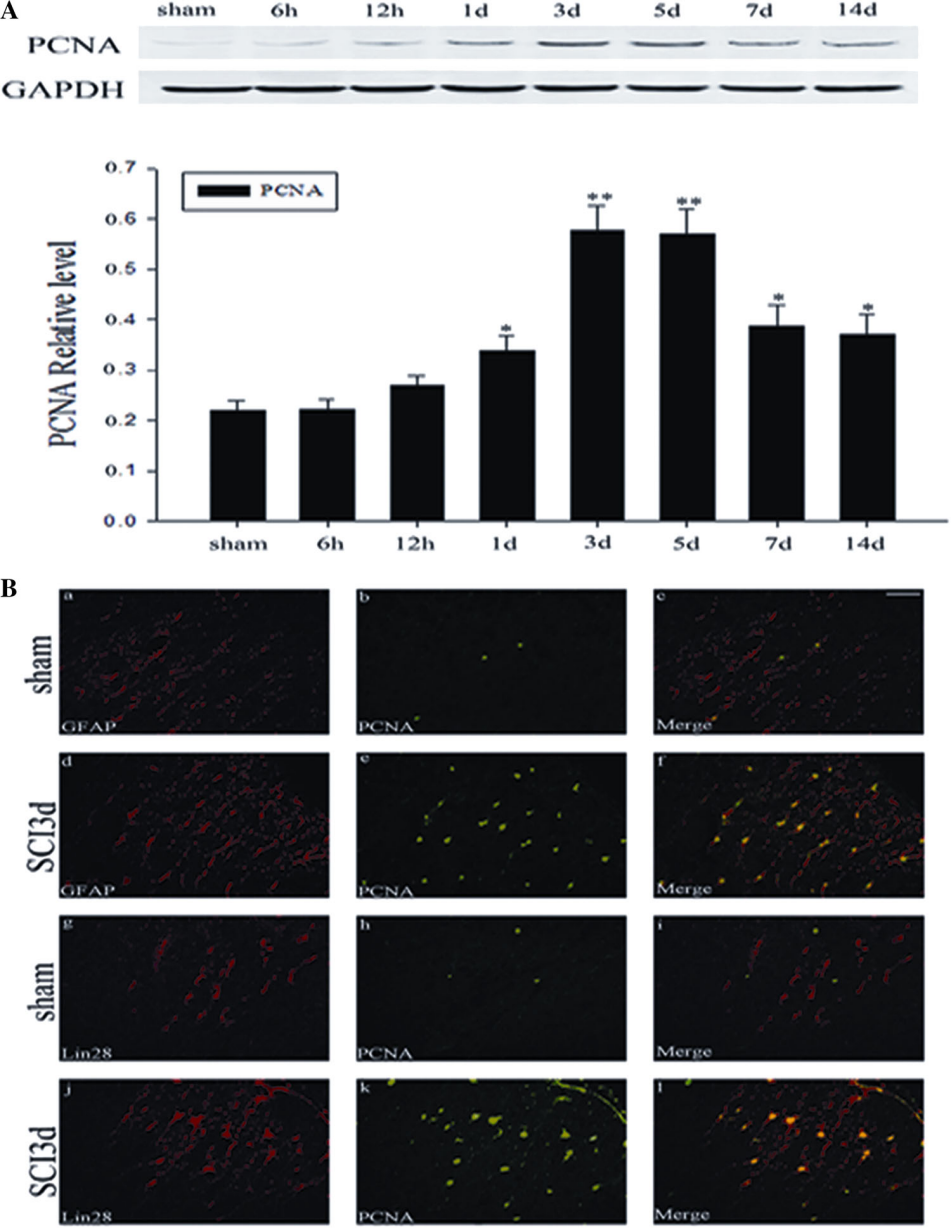
Association of LIN28 with the cell proliferation after SCI. Westernblot analysis of PCNA in spinal cord after SCI. The expression of PCNA was increased after SCI and peaked at day 3 (**A**). Double immunofluorescence staining is for PCNA, GFAP, and LIN28 in spinal cord after SCI (**B**). In adult spinal cord at 3 days after injury, sections labeled with PCNA (*e*) and GFAP (*d*) and the colocalization of PCNA with GFAP (yellow) were shown in the spinal cord (*f*). The majority of reactive astrocytes were PCNA-positive at 3 days after SCI (*f*). Moreover, there was colocalization between LIN28 and PCNA (l). However, we observed hardly any expression of PCNA in sham groups (*b*, *h*). *Scale bars*, 20 μm (*b*)

The following studies were performed to demonstrate the relationship between LIN28 expression and cell proliferation in the spinal cord after SCI. We first examined the expression of PCNA, which has been used as a general marker of dividing cells, in the injured spinal cord [44, 45]. PCNA expression increased gradually and reached a peak at 3–5 days and deceased thereafter (Fig. 6a). We next performed double-labeling immunofluorescent staining of PCNA together with GFAP or with LIN28 in SCI and sham spinal cords (Fig. 6b). As shown in (Fig. 6Bf), the majority of activated astrocytes were PCNA-positive. Furthermore, there was also colocalization between LIN28 and PCNA (Fig. 6Bi,l). However, almost no detectable expression of PCNA could be observed in the sham groups by immunofluorescent staining (Fig. 6Bb,h).

### The Expression and Correlation of LIN28 and NF-κB Signaling Pathway During LPS Induced Astrocytes Activation

It is well known that NF-κB signaling pathway plays an important role in the regulation of astrocytes inflammation. Sequence analysis revealed a highly conserved NF-κB motif in the first intron of the lin-28B gene [46]. In order to explore the expression pattern and possible mechanisms of LIN28 during astrocyte activation, we employed LPS (1 μg/ml), a widely used immunological stimulus, to mimic astrocyte activation in vitro [47]. As expected, the expression of iNOS, a marker of astrocyte activation, was gradually up-regulated 4 h post LPS treatment, reached a peak at 6 h, and then slightly declined.

**Fig. 7:**
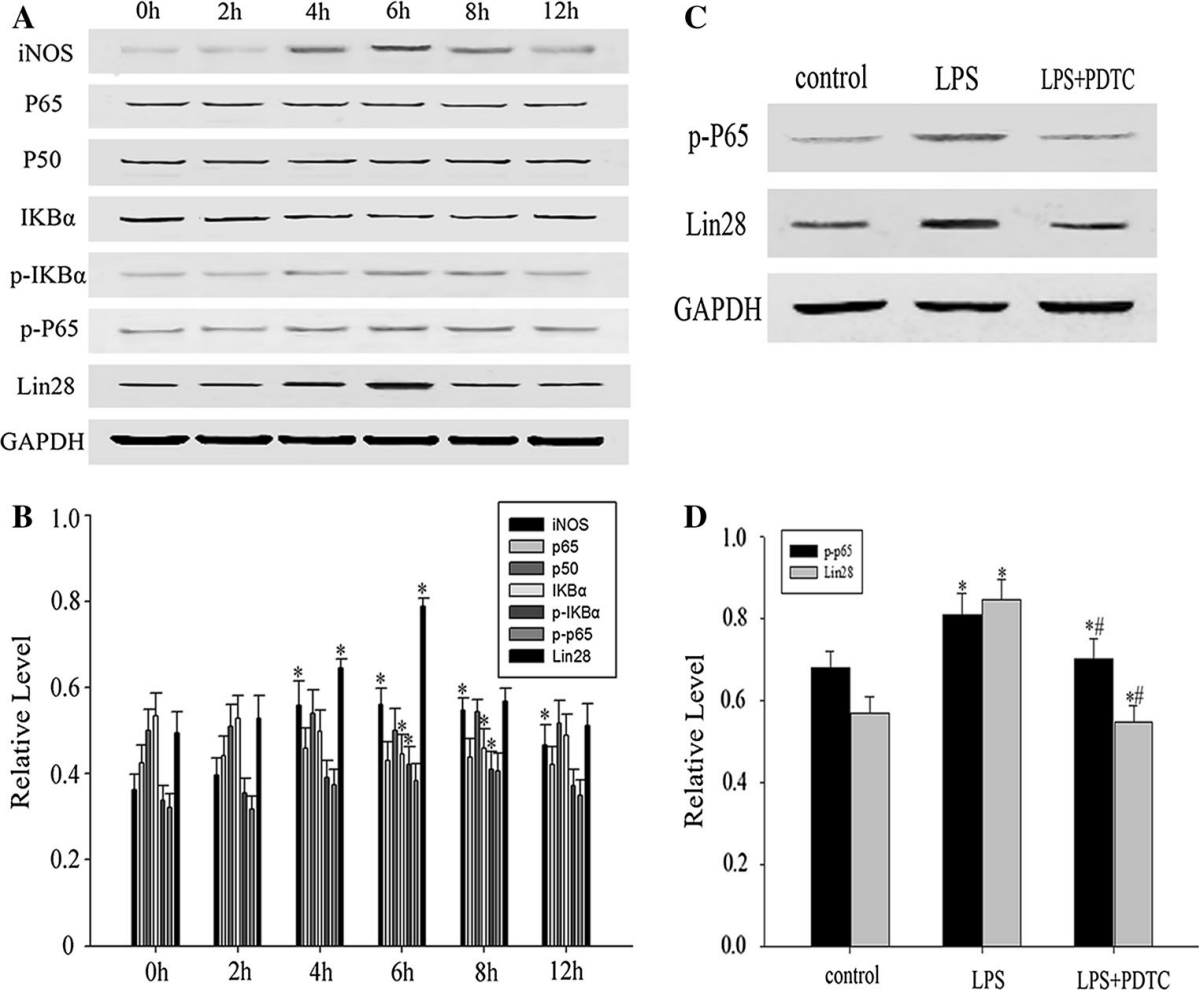
The expression and correlation of LIN28 and NF-κB signaling pathway during LPS induced astrocytes activation. Astrocytes were treated with LPS (1 μg/ml) for indicated times. INOS, p65, p50, IκBα, p-IκBα, p-p65, LIN28, and GAPDH expression was determined by Western blot analysis (**a**). Quantification of INOS, p65, p50, IκBα, p-IκBα, p-p65, and LIN28 protein levels. (n = 3, *p < 0.05) (**b**). The p-p65,LIN28 expression was analyzed by Western blot. Astrocytes in the presence or absence of LPS (1 μg/ml) or PDTC (100 uM) for 6 h were analyzed (**c**). Quantification of p-p65,LIN28 protein levels. (n = 3, *p < 0.05) (**d**)

Western blot detected a significant increased phosphorylation and a rapid protein degradation of IκBα in the astrocytes at 6 and 8 h post LPS treatment. Meanwhile, LIN28 protein expression was up-regulated by LPS stimulation, and the maximum response was observed at 6 h following LPS exposure (*p < 0.05) (Fig. 7a, b). Phosphorylation of p65 potentiates NF-κB transactivation [20]. Western blot analysis revealed an elevated phosphorylation level of p65 protein (p-p65) 6 h following LPS stimulation (Fig. 7a, b) (*p < 0.05). After pretreatment with PDTC, an NF-κB inhibitor, the LPS-induced p-p65 expression was significantly inhibited in astrocytes. Interestingly, blocking of NF-κB activation by PDTC greatly aborted LPS-induced expression of LIN28 (Fig. 7c, d). Taken together, our results demonstrated that LPS can stimulate LIN28 expression in astrocytes via the NF-κB signaling pathway.

## Discussion

The pathological process of SCI involves both primary and secondary injury mechanisms [48, 49]. It leads to a series of molecular and cellular events, which evolves over the following hours and days, resulting in reactive gliosis, neuronal apoptosis, inflammation, and oxidative stress [5, 50, 51]. These factors cause hypertrophy, proliferation, and migration of astrocytes [52, 53]. Astrocytes contribute to the formation of a glial scar at the lesion border, which functions as a physical and chemical barrier to axonal regeneration [54–58]. Astrocyte activation is required for effective immune responses in the central neuronal system [59, 60]. Deregulation of inflammatory responses by astrocytes has been suggested to participate in the development of several neurodegenerative diseases, including Alzheimer’s disease, Parkinson’s disease, HIV-associated dementia, and multiple sclerosis [61–63]. Despite great efforts which have been devoted to this field, the exact molecular mechanisms of SCI, especially the contributions of asctrocytes to SCI progression, have not been clearly understood. In this study, we employed a spinal cord injury model in adult rats and explored the potential molecular mechanisms after SCI.

LIN28 is a conserved regulator of cell fate succession in animals [64]. Previous reports demonstrated that LIN-28 is one of several factors that participate in reprogramming mammalian somatic cells to pluripotent cells and that regulate processes of the germline, postnatal development, and cancer occurances [22, 65, 66]. Here, we revealed the expression profiles of LIN28 in adult rat’s spinal cord after injury. Western blot analysis showed that the expression of LIN28 was significantly increased and peaked at 3 days after injury. Laser scanning microscopic observation detected LIN28 mainly expression in neuron and astrocyte. After SCI, LIN28 expression was greatly increased in activated astrocyte, but exerted no detectable change in neurons. Additionally, we also found that LIN28 was colocalized with PCNA, a general marker of dividing cells. Thus, SCI might enhance the expression of LIN28 in the activated astrocytes. These findings might provide important information to discuss the molecular and cellular mechanisms underlying the neuronal inflammation after SCI.

To further study the expression and possible function of LIN28 in astrocyte-mediated neuronal inflammation, we imitated astrocyte activation with LPS treatment in vitro. Amount of evidence supported the indispensible contribution of NF-κB signaling pathway to LPS-mediated astrocyte activation. Furthermore, some studies proved that such a process might involve LIN28 [46]. To test whether the relationship mentioned above exists in astrocytes, we determined the patterns of NF-κB and LIN28 expression in cultured primary astrocytes following LPS exposure. Using Western blot analysis, we examined the related molecular variation trend about NF-κB signaling pathway, including P65, P50, IκBα, p-IκBα, p-P65, and confirmed the activation of NF-κB signaling pathway in astrocytes following LPS stimulation. At the same time, LIN28 protein expressed in a time-dependent manner. Moreover, blocking NF-κB activation with PDTC, a NF-κB inhibitor, significantly inhibited expression of LIN28. Thus, the above data suggested that LIN28 was regulated by NF-κB signaling pathway in LPS-induced astrocytes activation. Taken together, this may enable us to have a further investigation on the cell level to elucidate these mechanisms.

Overall SCI induces recruitment of hematogenous cells to the injured site. These cells produce inflammatory cytokines such as interleukin (IL)-6, (IL)-1β, and TNF-α. And a lot of the literature demonstrated that astrocytes can be activated by LPS of appropriate dose/duration. These astrocytes reduces the production of pro-inflammatory cytokines IL-1β, IL-6 and TNF- α by interfering with TLR4/NF-κB signaling. These allow us to better combine vitro and in vivo experiments to discuss whether Influencing the LIN28 activation would be beneficial for astrocyte-mediated inflammation. In our view, since LIN28 through NF-κB signaling pathways involved in astrocyte inflammation of the SCI, then the expression of LIN28 by properly intervention would be helpful for CNS inflammation.

In summary, we investigated the protein expression and cellular localization of LIN28 during SCI. Our results provided a novel molecular pathway to detect the endogenous responses of CNS after SCI and a novel strategy for the treatment of spinal cord injury. Moreover, we found that LIN28 might involve in the activation of astrocytes following acute SCI. The results suggest that LIN28 is a useful role in controlling neuroinflammation. A further study should be performed to confirm the intrinsic mechanisms and functions of LIN28 in central nervous system injury and repair.

## Abbreviations

SCI: Spinal cord contusion injury
PCNA: Proliferating cell nuclear antigen
LPS: Lipopolysaccharide
CNS: Central nervous system
